# An Audit and Re Audit of Prelithium Workup in Psychiatry Inpatient Unit of Allied Hospital 2, Faisalabad

**DOI:** 10.1192/bjo.2026.11572

**Published:** 2026-06-30

**Authors:** Imtiaz Ahmad Dogar, Palvisha Sajid, Dua Fatima, Yahya Anwaar, Sadaf Ghulam Jelani

**Affiliations:** Faisalabad Medical University, Faisalabad, Pakistan

## Abstract

**Aims:**

To evaluate whether the pre-lithium workup in patients with Bipolar Affective Disorder (BPAD) initiated on lithium in Psychiatry in patient unit of Allied Hospital, Faisalabad adheres to the Maudsley prescribing guidelines in psychiatry.

**Methods:**

This study was conducted in 2025 at a tertiary care hospital in Faisalabad, data was collected from files of 72 inpatients in psychiatry ward prescribed lithium, to check if adequate pre lithium workup was done for each patient as recommended by Maudsley prescribing guidelines. A baseline audit was followed by a faculty led presentation on guidelines, and a re audit 3 months later, in which data was observed from 86 patients files. Initial findings revealed unsatisfactory workup . Results were discussed in a departmental meeting, leading to targeted teaching session for all the residents. The reaudit showed significant improvement in workup practices prior to prescribing lithium as recommended

**Results:**

The initial audit revealed that only 50(69.4%) out of 72 patients had their LFTs done, 3 (4.2%) had TFTs, 14(19.4%) had ECG and None of the patients got their weight measured before prescribing lithium.

A reaudit done 3 months later showed results from 86 patients according to which there was much improvement in compliance with the guidelines as 74(86%) had their LFTs, 53 (61.6%) had TFTs, 58 (67.4%) had ECG and 33 (38.4%) had weight measured before prescribing lithium.

These findings indicate a positive staff response and significant progress in adhering to pre lithium workup guidelines

**Conclusion:**

Lithium is the most effective mood stabiliser for the treatment and long-term prophylaxis of BPAD and helps in reducing relapse and suicide risk. Although it is very effective, lithium has a narrow therapeutic index and can cause range of adverse effects involving renal, thyroid, cardiovascular and metabolic systems. Therefore, it is essential to do baseline physical health assessment before initiating lithium therapy. It helps to identify pre-existing risk factors and ensures safe prescribing. Lithium can commonly cause hypothyroidism, renal impairment, weight gain and cardiac conduction abnormalities. The risk increases with longer treatment duration and cumulative exposure.

This audit demonstrated initially poor compliance with pre lithium workup as recommended by Maudsley prescribing guidelines. Rates of RFTs, TFTs, ECG and Weight measurement were very low which could have resulted in avoidable complications. Later intervention and reaudit showed much improved compliance with recommended standards. This highlights the importance of audit driven interventions leading to improvement in quality of care for patients in their best interest. Ongoing education, standardised pre-lithium checklists and regular re-auditing are recommended to ensure sustained compliance and further improvement in patient safety

